# Deep learning-based identification of chronic pulmonary embolism on CTPA: a regional lung analysis using multiplanar MIP images

**DOI:** 10.1186/s41747-026-00699-x

**Published:** 2026-03-16

**Authors:** Tuomas Vainio, Teemu Mäkelä, Arttu Ruohola, Anssi Arkko, Sauli Savolainen, Marko Kangasniemi

**Affiliations:** 1 HUS Diagnostic Center, Radiology, University of Helsinki and Helsinki University Hospital, Helsinki, Finland; 2 Department of Physics, University of Helsinki, Helsinki, Finland

**Keywords:** Deep learning, Hypertension (pulmonary), Lung volume measurements, Pulmonary embolism, Tomography (x-ray computed)

## Abstract

**Objective:**

Chronic pulmonary embolism (CPE) and chronic thromboembolic pulmonary hypertension (CTEPH) are challenging to diagnose, with delayed detection increasing mortality. We evaluated the performance of a convolutional neural network (CNN) in identifying these conditions from computed tomography pulmonary angiography (CTPA)-derived maximum intensity projection (MIP) images using a novel approach including proximal pulmonary vessels and a layered segmentation of the lung volume to assess the diagnostic value of different vascular regions.

**Materials and methods:**

We included 41 CPE, 41 acute pulmonary embolism (APE) and 41 normal controls (non-PE). 25 of the CPE patients had CTEPH confirmed by right heart catheterization. CNN classifiers were trained to identify CPE or CTEPH against a combined APE and non-PE group. Eleven masking schemes were applied for both classification tasks, resulting in 22 experiments. Model performances were compared using areas under the receiver operating characteristic curves (AUROC).

**Results:**

The model achieved good performance in distinguishing CPE from non-PE and APE cases (cross-validation AUROC 0.80) using full lung volume MIPs, while performance decreased with reduced data. For CTEPH classification against non-PE and APE, the model reached AUROC 0.88 with full data and 0.86 using only the most proximal half of the lung volume, suggesting key diagnostic features reside centrally. Using an open-source segmentation model, which excludes proximal vessels, resulted in lower AUROCs (0.74 for CPE, 0.83 for CTEPH).

**Conclusion:**

The cross-validation indicated that CPE and CTEPH could be identified from CTPA-derived MIP images, with performance improving as more vessels were included. The proximal vessels were most relevant for CTEPH detection.

**Relevance statement:**

Our study shows that neural networks can identify chronic pulmonary embolism in CTPA and the role of different vascular regions in that task, with the potential to improve future imaging diagnostics in patients with chronic pulmonary embolism.

**Key Points:**

- A convolutional neural network detects chronic thromboembolic pulmonary hypertension and chronic embolism from CTPA MIP projections.
- CTPA data were divided into four concentric anatomic layers for regional analysis.
- Central layers were most important for identifying CTEPH features.
- Network performance improved when more vessel regions were used as input.

**Graphical Abstract:**

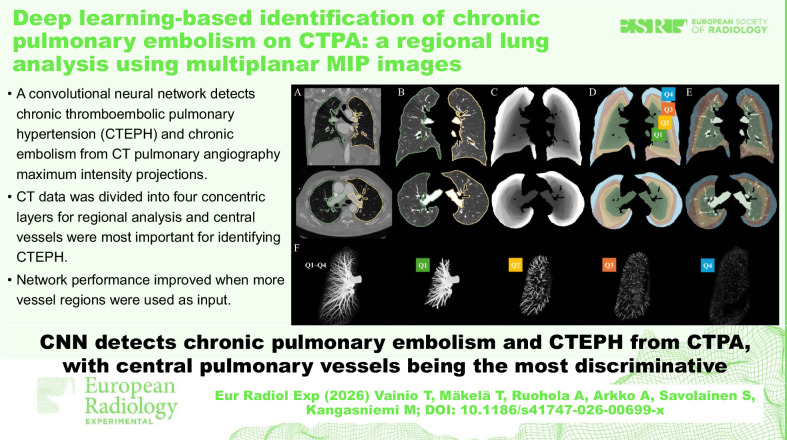

## Background

Chronic thromboembolic pulmonary hypertension (CTEPH) is a form of pulmonary hypertension resulting from unresolved thrombi after pulmonary embolism (PE) [1–4]. Although potentially curable through pulmonary endarterectomy, untreated CTEPH has a poor prognosis, emphasizing the importance of early and accurate diagnosis [5–7]. Diagnosis is often delayed due to the subtle and frequently missed radiologic signs on computed tomography pulmonary angiography (CTPA) [8, 9], including vessel narrowing, intimal irregularities, and mosaic perfusion patterns [10–12].

Many patients present with chronic pulmonary embolism (CPE) without meeting the hemodynamic criteria for CTEPH. These cases may represent an earlier or milder manifestation of the same disease spectrum and often remain underdiagnosed due to subtle imaging findings [13, 14]. The overlapping radiologic features between CPE and CTEPH further complicate detection, underlining the need for advanced diagnostic tools, such as artificial intelligence-based approaches.

Previous studies have demonstrated the potential of deep learning methods for detecting and classifying acute and chronic pulmonary embolism from CTPA images [15–19]. Full three-dimensional (3D) deep learning approaches can not only identify chronic thrombi or hypoperfusion associated with CPE, but also automatically extract pulmonary and cardiac morphometric features, classify CPE and CTEPH based on these measurements, and quantify disease severity [20, 21]. In contrast, our previous approach utilizes two-dimensional (2D) maximum intensity projection (MIP) images derived from CTPA, allowing the network to focus more directly on the overall morphology of the pulmonary vasculature, while reducing computational demands compared to full 3D models [19]. A limitation of the previous study was the exclusion of the most proximal pulmonary vessels, which are often affected in CTEPH by features such as vessel dilatation, tortuosity, and wall irregularities [22–25]. However, their specific contribution remains relatively understudied.

In our current study, we present a novel lung segmentation strategy that divides the lungs into four volumetrically equal layers, progressing from the central vasculature toward the periphery. The first layer includes the most proximal 25% of the lung volume, starting from the base of the large pulmonary vessels, followed by the next proximal 25%, with the final two layers representing the most distal 25% each. This approach enables systematic analysis of which anatomical regions contribute most to the classification of CPE and CTEPH.

Our goal was to investigate how different vascular regions contribute to the performance of a convolutional neural network (CNN)-based classifier in detecting CPE and CTEPH. Given the limited size of the local dataset, we applied cross-validation to compare different masking schemes. Our main hypothesis was that including proximal pulmonary vessels in the input data would enhance performance. Our approach provides new insights into the radiologic features most relevant for identifying CPE and CTEPH, with the potential to enhance early diagnosis and improve clinical outcomes.

## Materials and methods

### Subjects

The cohort in this study is an expansion of our previous study cohort [19] with additional patients. The original retrospective cohort included 26 patients with CPE, 26 with acute pulmonary embolism (APE) and 26 with no PE retrospectively identified from the Helsinki University Hospital district picture archiving and communication system‒PACS between 2017 and 2019 based on ventilation–perfusion scans and CTPA reports.

For the current study, the original patients were retained, and additional cases were identified using updated inclusion criteria made possible by the hospital PACS.

In the updated inclusion criteria, CPE patients were identified from CT scans performed between January 1, 2021, and June 30, 2023, using the “chronic pulmonary embolism” protocol. Patients were included if radiological findings were consistent with chronic pulmonary embolism and not questioned in clinical notes or subsequent reports. If multiple CTPAs were available, the first scan with CPE findings was selected.

“Non-PE” patients were identified from the same imaging protocol and time frame. Patients were included if they showed no radiological signs of APE or CPE.

Acute PE patients were identified from monoenergy CTPAs performed between January 1 and September 9, 2021. Patients were included if radiological findings were consistent with APE, with no signs of CPE, and no diagnosis of CPE or death within 2 years after the scan. A CTEPH subgroup was derived from the CPE group by selecting patients in whom CTEPH was confirmed by right heart catheterization.

**Fig. 1:**
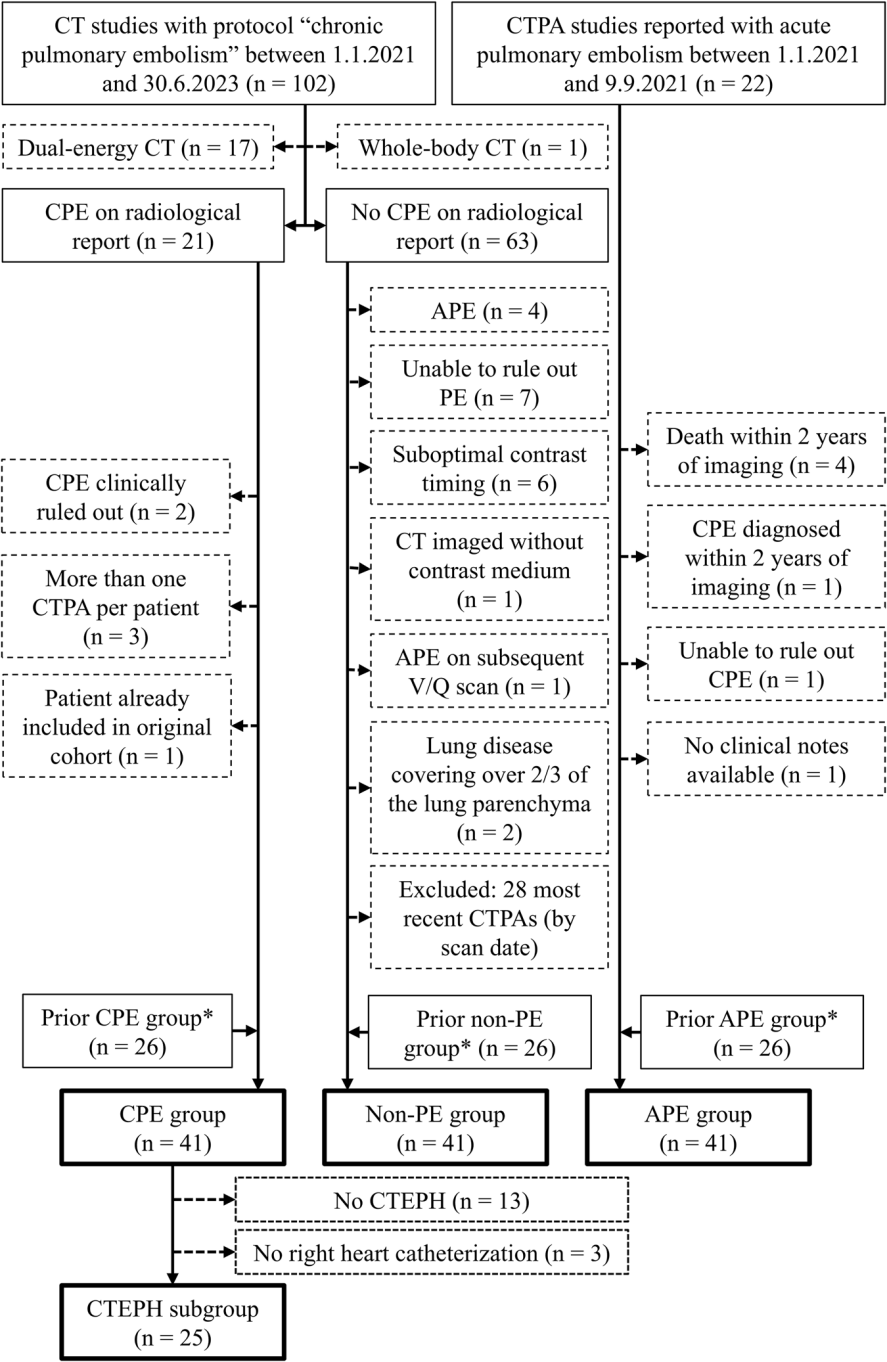
Flowchart illustrating patient selection and allocation into study groups and subgroups. APE, Acute pulmonary embolism; CPE, Chronic pulmonary embolism; CT, Computed tomography; CTEPH, Chronic thromboembolic pulmonary hypertension; CTPA, Computed tomography pulmonary angiography; PE, Pulmonary embolism; V/Q scan, Ventilation–perfusion scintigraphy. * Selection criteria for the prior CPE, non-PE, and APE groups are described in our earlier work [19]

In total, we had 41 CPE, 41 APE, and 41 non-PE patients who met the inclusion criteria. 25 patients with CTEPH were diagnosed through right heart catheterization and included in the CTEPH subgroup. Three CTEPH patients diagnosed only by echocardiography were excluded from the CTEPH subgroup. The inclusion flowchart is shown in Fig. 1, patient characteristics in Table 1, and the CT scanners and the x-ray tube voltages are shown in Table 2.

**Table 1:** Patient characteristics

Subgroup	Age, median (min–max)	Females	Males	CTEPH	RV/LV ≥ 1	RV/LV < 1	ePAP
Chronic PE	64 (19–82)	26	15	28	32	9	1
No PE	65 (28–88)	22	19	0	16	25	8
Acute PE	63 (29–90)	23	18	0	15	26	4
All	63 (19–90)	71	52	28	63	60	13

*CTEPH* Chronic thromboembolic pulmonary hypertension, *ePAP* Elevated pulmonary arterial pressure due to other causes than CTEPH, *PE* Pulmonary embolism, *RV/LV* Right ventricle/left ventricle ratio

**Table 2:** CT-scanner models used in the study

Scanner (manufacturer)	x-ray tube voltage							Total
	70 kV	80 kV	90 kV	100 kV	110 kV	120 kV	140 kV	
Aquillon Prime (Toshiba)	0	0	0	2	0	0	0	2
Somatom Definition AS (Siemens)	0	0	0	5	0	6	0	11
Somatom Definition AS+ (Siemens)	0	0	0	5	0	0	0	5
Discovery (GE)	0	0	0	3	0	0	0	3
Discovery HD (GE)	0	0	0	1	0	0	0	1
Somatom Edge (Siemens)	0	4	0	27	0	9	1	41
Somatom Flash (Siemens)	0	0	0	10	0	2	0	12
Somatom Force (Siemens)	1	0	5	1	1	7	0	15
Lightspeed VCT (GE)	0	0	0	10	0	1	0	11
Revolution EVO (GE)	0	0	0	9	0	4	0	13
Revolution HD (GE)	0	1	0	5	0	0	1	7
Somatom go.TOP (Siemens)	0	1	0	1	0	0	0	2
Total	1	6	5	79	1	29	2	123

### Data preprocessing

For classification, we converted the 3D CTPA volumes into sets of 2D MIP images. These were then used for neural network training. The use of MIPs required the surrounding high attenuation structures, such as bones, mediastinum, trachea, main bronchi, heart, great vessels, and hila, to be excluded by performing lung segmentation.

The Digital Imaging and Communications in Medicine‒DICOM volumes were first converted to Neuroimaging Informatics Technology Initiative‒NIfTI file format using *dcm2niix* tool [26]. An initial segmentation for the left and right lungs, which included smaller vessels and bronchi, was achieved by a deep learning-based tool, *lungmask* 0.2.8, from Hofmanninger et al [27]. However, since the proximal pulmonary vessels in the hila and mediastinum were missing in the segmentations, they were manually segmented back into the CT data using the image processing platform 3D Slicer [28]. A radiologist with over 5 years of experience in vascular imaging segmented the roots of the right and left pulmonary arteries from their bifurcation points, as well as the roots of the pulmonary veins from their junctions with the left atrium. Subsequently, a threshold-based tool was used to automatically segment the remaining portions of the vessels from these roots toward the periphery. The segmentations were then manually corrected and refined by a radiologist, a research scientist, and a medical physicist, and all final segmentations were reviewed and verified by the radiologist. Finally, these proximal vessels were included in the initial segmentation, producing the final full segmentation.

**Fig. 2:**
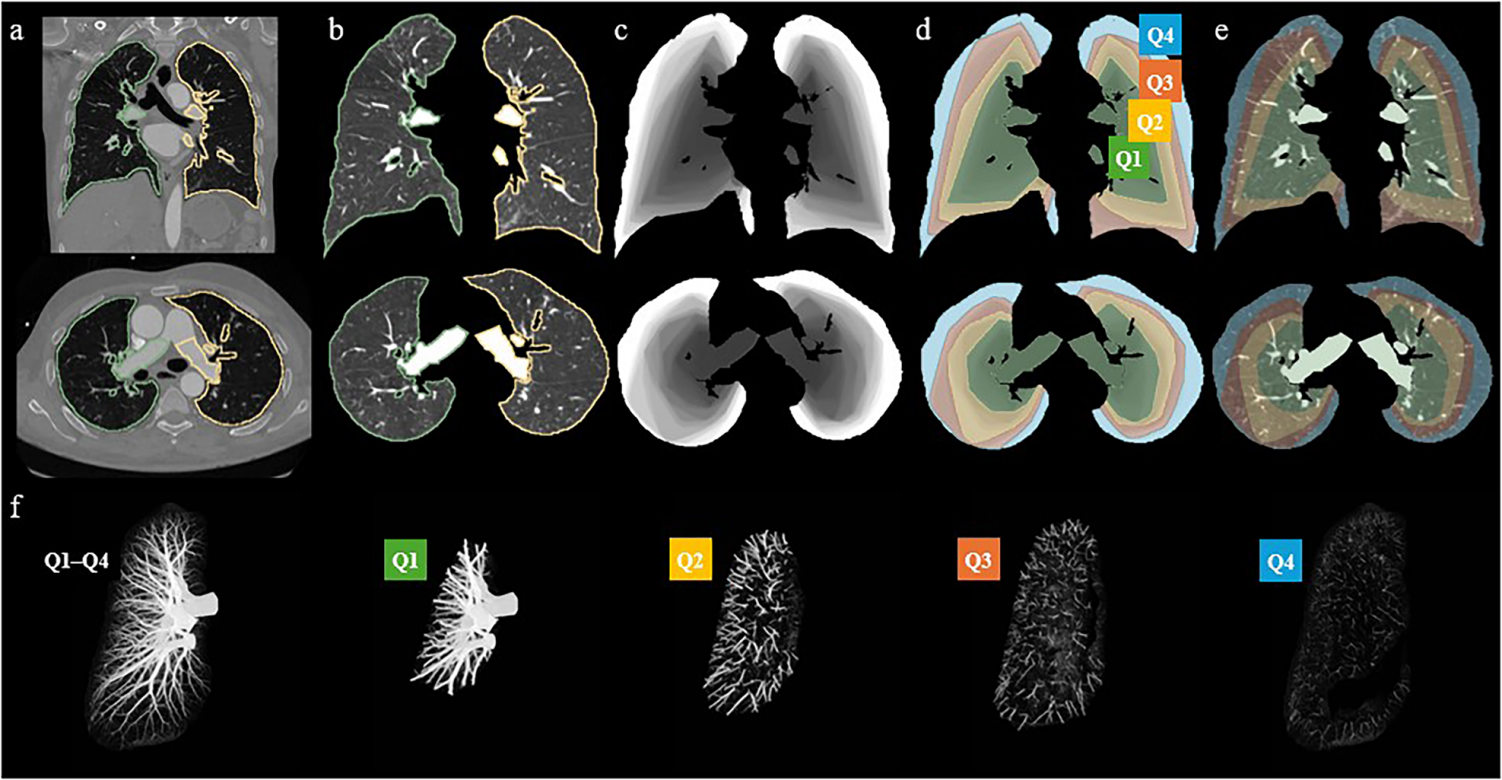
Illustration of the different lung segmentation schemes. Initial lung masks (**a**, **b**; green and yellow outline) were created for the computed tomography pulmonary angiography volume. The masks were divided into four volumetrically equal layers (**d**–**f**; Q1, Q2, Q3, and Q4), extending from the central regions to the periphery (**c**). This approach allowed the lungs and pulmonary vessels to be provided in different combinations to the neural networks for classification performance analysis

The segmented lung volume was divided into four layers with equal volumes (four quarters, Q1–Q4), starting from the central region near the hilum and extending outward to the periphery (see Fig. 2). Due to the complexity and variability in lung shapes, automatic classification into systematic layers starting from the center and ending in the periphery is a nontrivial task. To achieve this, we developed an iterative algorithm to create a volume with voxel values ordered from the central vessels to the periphery. The algorithm started by identifying voxels with the highest attenuation within the manually corrected segmentation, which we ensured were in the central vessels. Subsequently, the inclusion threshold was incrementally decreased by one HU, and the new voxel regions exceeding each new threshold were labeled consecutively. A convex closing algorithm was applied at each threshold step to incorporate nearby low-attenuation parenchymal regions inside the full mask. The ordered voxel values were then normalized to a 0–1 range to correspond to the fraction of the total lung volume starting from the central vessels. The quartile region masks (Q1, Q2, Q3, and Q4) could thereafter be obtained by using 0.25 increments in threshold values. We repeated this procedure separately for the left and right lungs of each patient.

We used various combinations of Q1, Q2, Q3, and Q4 masks to create different sets of MIP images. Projections were generated for the innermost layer (Q1) alone and progressively larger combinations including the adjacent layers (Q1–Q2, Q1–Q3, Q1–Q4). Additionally, MIPs were generated for combinations excluding the innermost layers (*i.e*., only include Q2–Q4, Q3–Q4). Individual sets of MIPs were also created for each of the four layers alone. This approach enabled a comprehensive assessment of the contribution of different vascular structures by visualizing them in different concentric zones. A set of MIPs was also created using the initial *lungmask* segmentation without the manually added proximal vessels.

As one of our primary objectives was to determine whether the inclusion of proximal vessels impacts classification performance, we conducted two supplementary analyses of vessel sizes. The aim was to investigate whether the vessel dilation alone could indicate either CPE or CTEPH within our study population. We recorded two measurements for each patient: (1) the volume of the segmentations that were added manually to the initial segmentations, and (2) the pulmonary artery cross-sectional area. To emphasize the expected changes, the latter was the larger of the right and left pulmonary artery cross-sectional areas, measured near the bifurcation point and perpendicular to their axes. These were then used for classification to produce reference AUROC values.

### Neural network architecture and training

The neural network comprised a pretrained feature encoder CNN with frozen weights and a trainable classification tail. Eleven MIP images captured at standardized angles from either the left or right lung were used as the network input: starting from the anteroposterior view, the view was rotated around the left-to-right horizontal axis and separately around the vertical axis. The images were augmented so that these projection angles were randomly changed ± 3° from the baseline, and the 2D MIP images were likewise rotated randomly ± 3°.

Each grayscale MIP image (224 × 224 × 3, with three identical channels) was first passed through the DenseNet-121 model with pretrained ImageNet-1K weights from the *torchvision* library, followed by the classification layer. The latter consisted of the feature vector (1,024 elements) being passed through batch normalization, a fully connected layer (1,024 neurons), Alpha Dropout (drop probability 0.3), batch normalization, another fully connected layer (512 neurons), Alpha Dropout (0.3), batch normalization, and a final fully connected layer with one output neuron. Finally, the eleven predictions for each pass-through were averaged to produce a single value representing classification decisions: CPE *versus* non-CPE, or CTEPH *versus* non-CPE. Left and right lungs were treated separately during training with the same ground truth label.

In our experiments, we kept the neural network architecture fixed (no hyperparameter tuning) and varied only the input data. The network architecture motivation is detailed in our previous work [19]. We trained the network for 30 epochs in each experiment using the same batch size of eight, Adam optimizer with an initial learning rate of 0.0002 and weight decay of 0.001, cosine learning rate scheduling, and binary cross entropy loss.

Each experiment was performed ten times with different randomized folds. Each of these ten shuffles was performed using five-fold cross-validation/testing, and the training for each fold was repeated five times. Both lungs of each patient were always kept in the same cross-validation fold and never appeared in more than one-fold within a given split to prevent data leakage. The same classification label was assigned to both lungs within a single case. The split and fold randomizations were identical between the experiments. This resulted in ten independent runs for each experiment, as well as patient-level predictions taken as an average of the individual hold-out sets from the last epoch.

### Performance and statistical testing

We used the area under the receiver operating characteristic curve (AUROC) to evaluate and compare how the masking schemes affected neural network classification of CPE or CTEPH. In detail, an ensemble of AUROCs was calculated for each experiment described in the “Data preprocessing” and “Neural network architecture and training” sections. For each experiment, AUROC values were averaged over the folds and repeats, resulting in ten AUROC estimates (*i.e*., the ten shuffles) per configuration. Different segmentation schemes were subsequently compared with each other to assess how much the different lung regions affected the diagnostic accuracy of the model. The normality of the data within each group was assessed using the Shapiro–Wilk test. Homogeneity of variances was evaluated using Levene’s test. Friedman’s test was used to determine the presence of group differences. Pairwise Wilcoxon signed-rank test was used to assess the difference between the various segmentation combinations and the full segmentation (Q1–Q4). A *p*-value of < 0.05 was considered indicative of a significant difference in performance. The statistical tests were performed using SPSS Statistics software version 29.0.0.0 (IBM Corp).

### Visual assessment

CTPA images misclassified by the repeated model ensemble, either as false positives or false negatives, were reviewed by a radiologist. For each individual model, a conservative threshold of 0.5 was applied, maximum was taken from the left and right lung, and the final classification decision was determined through majority voting across the training repeats. The aim of this review was to determine if there were any specific features present that could explain the misclassification of the neural network. We sought to identify common characteristics or patterns, such as artifacts, anatomical variations, or indistinct vascular structures, that might have led to incorrect predictions.

## Results

### CNN performance

**Fig. 3:**
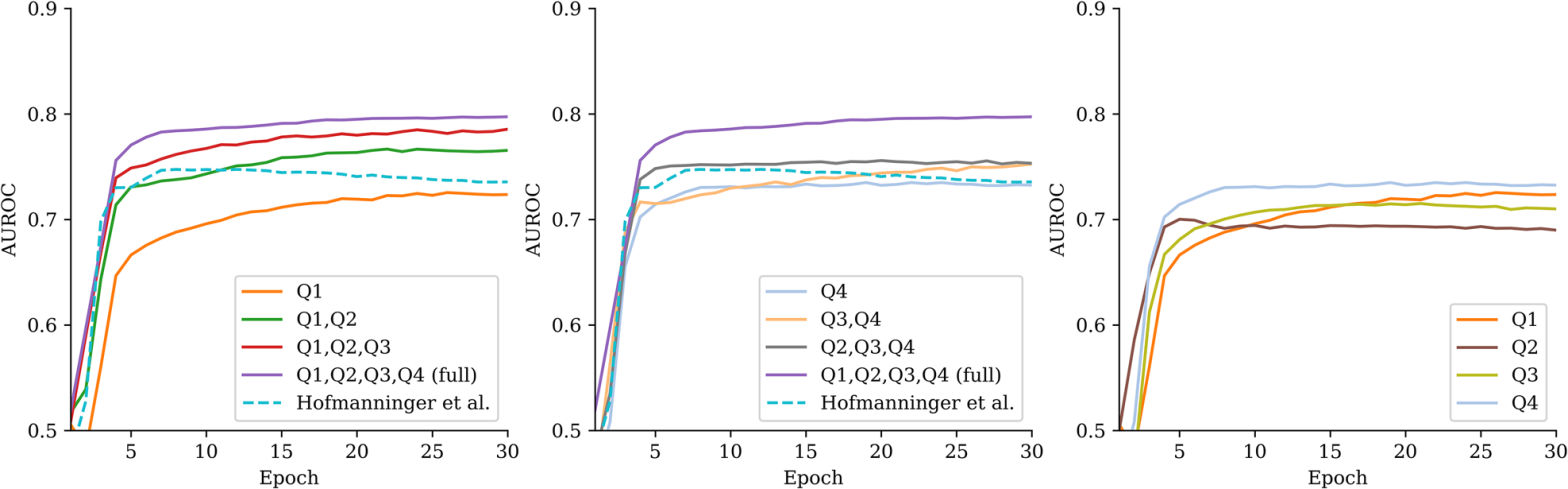
The graphs show the evaluation of the neural network cross-validation performance for classifying the chronic pulmonary embolism (CPE) group from the non-CPE group. Each curve represents the area under the receiver operating characteristic curve (AUROC) performance during training of different segmentation scheme. The best performance was achieved when the full computed tomography pulmonary angiography data (Q1–Q4) were used. Performance decreased gradually when data was removed either from the center (left graph) or the periphery (middle graph). The dashed line represents the initial reference segmentation in which the largest proximal vessels are missing. The right graph shows the model’s performance using only one of the volumetric quarters at a time, which all showed a similar performance

In binary classification between the CPE group and the combined non-PE and APE group (later referred to as non-CPE), the CNN had a good average cross-validation performance with AUROC 0.80 when analyzing the MIPs created from the whole data (Q1–Q4). The performance gradually worsened as layers of the lungs were removed from the input data, as seen in Table 3 and Fig. 3. All *p*-values reported in Table 3 represent comparisons against the full Q1–Q4 scheme. In comparison, classification performance was significantly lower with an AUROC of 0.74 when using the initial automatic lung segmentation by Hofmanninger et al [27].

**Table 3:** AUROC values for different segmentation schemes in CPE and CTEPH datasets

Segmentation	CPE *versus* non-CPE		CTEPH *versus* non-CPE	
	AUROC, mean (min–max)	*p*-value	AUROC, mean (min–max)	*p*-value
Full	0.80 (0.75–0.82)	NA	0.88 (0.86–0.90)	NA
Q1	0.72 (0.69–0.75)	0.005*	0.85 (0.82–0.88)	0.012
Q1, Q2	0.77 (0.72–0.78)	0.007	0.86 (0.83–0.90)	0.105
Q1, Q2, Q3	0.79 (0.73–0.81)	0.027	0.83 (0.81–0.86)	0.004*
Q2	0.69 (0.67–0.72)	0.005*	0.77 (0.75–0.79)	0.005*
Q2, Q3	0.70 (0.64–0.76)	0.005*	0.79 (0.74–0.81)	0.005*
Q2, Q3, Q4	0.75 (0.69–0.78)	0.005*	0.85 (0.83–0.87)	0.005*
Q3	0.71 (0.68–0.73)	0.005*	0.78 (0.74–0.81)	0.005*
Q3, Q4	0.75 (0.71–0.78)	0.005*	0.82 (0.78–0.86)	0.005*
Q4	0.73 (0.69–0.77)	0.005*	0.79 (0.76–0.83)	0.005*
Hofmanninger et al [27]	0.74 (0.70–0.77)	0.005*	0.83 (0.81–0.85)	0.005*

The table presents the mean and range of area under the receiving operator characteristic curve (AUROC) values for each segmentation scheme, both for the full chronic pulmonary embolism (CPE) dataset and the chronic thromboembolic pulmonary hypertension (CTEPH)-only subset. The *p*-values indicate whether the AUROC obtained from a given lung-layer combination differed significantly from the AUROC achieved using the full lung segmentation (Q1–Q4), with statistical significance (*) at *p* < 0.05/10. Q1 refers to the most central layer, while Q2, Q3, and Q4 denote increasingly distal layers

*NA* Not applicable

**Fig. 4:**
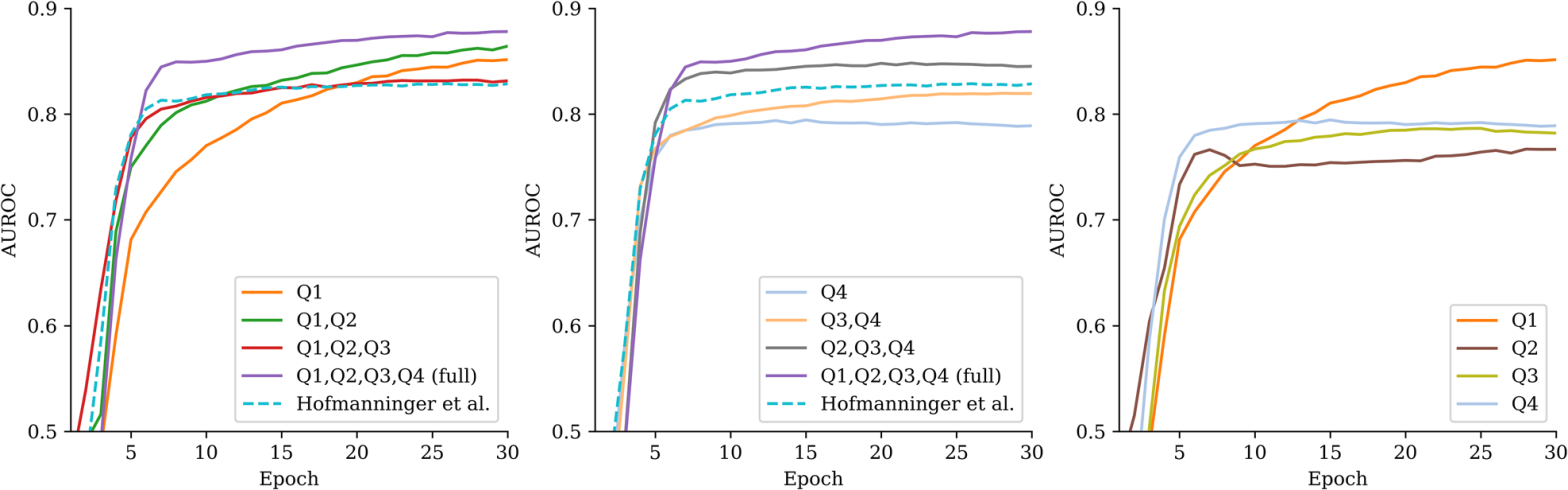
The graphs show the evaluation of the cross-validation performance for classifying the chronic thromboembolic pulmonary hypertension (CTEPH) subgroup from the non-CPE group. The best performance was achieved when the full CTPA data (Q1–Q4) was used. Performance declined when central (left graph) or peripheral (middle graph) parts of the data were removed. The dashed line represents the initial segmentation in which the largest proximal vessels are excluded. The right graph shows the performance when only one of the four volumetric quarters was used at a time, with the central quarter (Q1) showing the highest performance

In the classification of the CTEPH group against the non-CPE group, the model achieved a good performance with an AUROC of 0.88 when using the full data (Q1–Q4). As parts of the lung were removed from the analysis, the performance declined, and several segmentation schemes resulted in significantly lower AUROCs, see Table 3 right-hand side and Fig. 4. Notably, the segmentation including only the central lung regions (Q1–Q2) yielded an AUROC of 0.86, which was not significantly different from the full data. This suggests that most of the relevant diagnostic information for CTEPH is located centrally. In contrast, using only peripheral areas (Q3–Q4) resulted in a significantly lower performance with an AUROC of 0.82. When using the Hofmanninger et al lung segmentation method [27], the performance was good with an AUROC of 0.83 for CTEPH identification but was significantly lower than with full Q1–Q4 data.

### Pulmonary artery cross-sectional area and vascular volume measurements

We performed two measurements related to vascular sizes (manually added volumes and pulmonary artery cross-sectional areas) and compared the differences between APE, CPE, and non-PE groups and between APE, CTEPH, and non-PE (sub)groups. The pairwise comparison results are summarized in Table 4.

**Table 4:** Results from pulmonary artery cross-section area measurements and additional volume segmentations

Subset or comparison	Area (cm^2^)	Volume (mL)
APE, mean (± SD)	5.1 (± 1.3)	85.2 (± 22.7)
CPE, mean (± SD)	6.2 (± 1.8)	103.4 (± 35.4)
CTEPH, mean (± SD)	6.3 (± 1.4)	108.4 (± 31.3)
Non-PE, mean (± SD)	6.0 (± 2.3)	88.5 (± 29.3)
CPE *versus* APE, Δ (*p*-value)	+1.1 (0.007)*	+18.3 (0.020)
CPE *versus* non-PE, Δ (*p*-value)	+0.2 (0.409)	+14.9 (0.055)
CTEPH *versus* APE, Δ (*p*-value)	+1.2 (0.002)*	+23.2 (0.002)*
CTEPH *versus* non-PE, Δ (*p*-value)	+0.3 (0.197)	+19.9 (0.008)*
Non-PE *versus* APE, Δ (*p*-value)	+0.9 (0.157)	+3.4 (0.813)

*APE* Acute PE, *CPE* Chronic pulmonary embolism, *CTEPH* Chronic thromboembolic pulmonary hypertension, *PE* Pulmonary embolism, *SD* Standard deviation, *Δ* Difference in mean values

* Statistically significant (*p* < 0.05/3)

As the assumption of homogeneity of variance was violated in the pulmonary area comparisons based on Levene’s test (*p* = 0.008 or less), and at least one subgroup violated the assumption of normality based on the Shapiro–Wilk test in each area and volume comparisons (*p* = 0.046 or less), we performed group-level analyses using Kruskal–Wallis tests. Since each of the four investigations (two types of measurements, two subgroup comparisons) showed statistically significant differences in central tendencies (*p* = 0.046 or less), we conducted post-hoc pairwise analyses using the Mann–Whitney U test with Bonferroni correction, considering significance at *p* < 0.05/3 and concluded the following. We observed small (1 cm^2^) but statistically significant differences in pulmonary artery cross-sectional areas between CPE and APE (*p* = 0.007) and between CTEPH and APE subgroups (*p* = 0.002), but no statistically significant difference between CPE or CTEPH and non-PE subgroups. There were statistically significant differences in volume measurements between CTEPH and APE (*p* = 0.002) and between CTEPH and non-PE subgroups (*p* = 0.008), suggesting discriminative possibilities. We observed no other statistically significant differences.

We investigated the area and volume measurements’ capabilities for classifying between CPE (*n* = 41) and non-CPE (*n* = 82) and between CTEPH (*n* = 28) and non-CPE subgroups. For CPE classification, AUROC was 0.61 (95% CI: 0.51–0.72) for area and 0.64 (0.53–0.74) for volume measurements, indicating poor but significantly better performance than random guessing based on 0.5 being outside the CIs. Corresponding AUROCs for CTEPH classification were 0.66 (0.55–0.77) and 0.71 (0.60–0.81), indicating poor and moderate classification performance, respectively.

### Visual assessment

Using a conservative threshold of 0.5 at the final epoch and averaging the different training runs, the cross-validation ensemble model misclassified eight CPE, six APE, and thirteen non-PE patients in CPE classification. For CTEPH classification, two CTEPH, eight APE, and thirteen non-PE cases were misclassified. A radiologist reviewed all misclassified cases and found no consistent imaging features or artifacts explaining majority of misclassifications. Several misclassified cases overlapped between CPE and CTEPH detection.

Among the false-negative CPE cases, five patients had MIP image opacities from atelectasis (one case), hypoventilation (three cases), or both (one case). Two patients had only minimal CPE findings, and one patient had pleural plaques causing opacities in MIP. An RV/LV ratio greater than one was observed in four cases, although the heart was not visible in the input MIP images.

Among false-positive CPE cases, three had atelectasis. Vascular distortion was noted in six cases (two APE and four non-PE), caused by atelectasis, scarring, idiopathic pulmonary hypertension, missed chronic embolism, or abscess. One non-PE patient had peripheral vascular pruning from lung hyperinflation post-lobectomy. Three non-embolic cases had pulmonary hypertension due to Sjögren’s syndrome, congestive heart failure, or idiopathic pulmonary arterial hypertension, the latter with proximal pulmonary artery dilatation and mosaic perfusion. One APE patient had chronic embolism misinterpreted as acute embolism in the initial radiology report. Nine patients had pleural plaques. An RV/LV > 1 was observed in six non-PE and four APE cases.

In the false-negative CTEPH cases, one had suboptimal inspiration, while another had no identifiable reason for misclassification. One had RV/LV > 1.

Among false-positive CTEPH cases, six had atelectasis, three scarring, and one hypoventilation. Vascular distortion was observed in six cases (two APE and four non-PE) due to various causes. One non-PE patient had vascular pruning post-lobectomy. One APE patient had undiagnosed chronic embolism with typical findings, which were missed in the original report and patient selection. Among the non-PE cases, one had elevated pulmonary pressure in echocardiography due to congestive heart failure, but no other radiological signs of pulmonary hypertension except RV/LV > 1. Another non-PE patient with idiopathic pulmonary arterial hypertension showed dilated proximal pulmonary arteries, RV/LV > 1, and a mosaic perfusion pattern in CTPA. In total, RV/LV > 1 was present in five non-embolic cases and six APE cases.

The MIP images of the different lung segments were reviewed by the radiologist to determine obscuring factors such as atelectasis, hypoventilation, or imaging artifacts. Masking due to these factors impacted vessel visibility in 48 patients, affecting segment Q1 in 6 cases, Q2 in 32 cases, Q3 in 40 cases, and Q4 in 8 cases.

## Discussion

We evaluated several masking schemes as inputs for a CNN-based model aimed at detecting CPE and CTEPH on CTPA MIP images. When analyzing the entire pulmonary vascular tree, the CNN achieved average cross-validation AUROCs of 0.80 for distinguishing CPE from non-CPE and 0.88 for classifying CTEPH from non-CPE. These results are consistent with prior studies, which reported AUROCs ranging from 0.68 to 0.94 [18].

We segmented the CTPA volumes into four equally sized layers, structured concentrically from the central regions toward the periphery. Systematic exclusion of vascular regions showed that all regions contributed to model performance, which improved with more extensive input. The only exception was a slight AUROC decrease for CTEPH when adding Q3 to Q1–Q2, likely due to imaging noise; this effect was not seen in CPE.

The AUROC values of different individual vascular regions (Q1, Q2, Q3, Q4 separately) were close to each other in CPE diagnosis. However, in CTEPH diagnosis, the innermost quarter (Q1) demonstrated a higher AUROC than the other quarters and even outperformed the combined AUROC of the remaining regions (Q2–Q4). This suggests that while incorporating additional regions improved overall performance, no single vascular region contributed disproportionately to diagnostic accuracy in CPE. In contrast, the most central vascular region played a crucial role in CTEPH diagnosis, and there was no statistically significant difference in the performance whether the identification of CTEPH was based on the two most central segments (Q1–Q2) or the full vascular data. While this finding was somewhat unexpected, it is nevertheless plausible given that pulmonary hypertension leads to increased upstream pressure, causing dilatation and tortuosity of the proximal pulmonary vessels.

Similar vascular changes are also observed in other forms of pulmonary hypertension, which posed a challenge for the CNN’s performance. Some false-positive cases had pulmonary hypertension of other etiologies (*e.g*., Sjögren’s disease, idiopathic pulmonary arterial hypertension, congestive heart failure). Nonetheless, most such controls were correctly classified, suggesting the CNN captured features specific to CTEPH rather than general pulmonary hypertension.

The volumetric analysis of proximal vessels indicates that proximal vessel dilation alone did not account for the model’s performance. Vessel volumes did not differ significantly between CPE and controls, and although CTEPH volumes were higher than controls, the resulting AUROCs (0.64 and 0.71) were notably lower than those of the CNN (0.80 and 0.88, respectively).

Our study included 25 CTEPH patients confirmed by right heart catheterization and introduced a novel regional segmentation approach. However, certain limitations should be acknowledged.

The first limitation is the retrospective design of our study and possible selection bias due to variability in reporting radiologists. To minimize selection errors, we excluded APE patients later diagnosed with CPE or deceased within 2 years after CTPA. Nevertheless, one APE case showed clear signs of CPE upon retrospective review. This likely affected network performance but also highlights its ability to detect a case initially misinterpreted by the radiologist.

Second, some misclassified cases showed parenchymal or vascular abnormalities that may have influenced CNN performance. Although the visibility of pulmonary vessels affected by atelectasis or hypoventilation was recorded, a systematic assessment of the prevalence of all such confounding abnormalities was not performed and represents an area for future investigation. The clinical severity of CPE/CTEPH was not assessed beyond whether pulmonary hypertension was present, and future studies should investigate the correlation between CNN performance and CTEPH severity. In addition, our study did not evaluate the CNN’s ability to distinguish CTEPH from CPE without pulmonary hypertension, due to the limited number of patients with hemodynamically confirmed pulmonary hypertension, which should also be addressed in future research.

Due to partly manual lung segmentation, observer bias cannot be fully excluded. The segmentation also excluded the heart and systemic veins, potentially leaving out relevant diagnostic information. Furthermore, MIP images are susceptible to motion and density artifacts that may affect vessel appearance. Future work should aim for fully automated segmentation to improve vessel extraction and reduce artifact-related errors.

Given the limited size of the dataset, we opted not to use a separate test set in order to maximize the number of training cases. Since our primary goal was to evaluate relative differences (or potential superiority) between the masking schemes, we employed multiple repetitions of cross-validation combined with a threshold-free AUROC approach to reduce the impact of random variation. To address the limitations of data size and confirm the generalizability of these findings, a multi-center study incorporating independent external test cohorts would be an important next step.

In conclusion, the CNN- and MIP-based approach demonstrated promising capability in detecting CPE and CTEPH, achieving strong cross-validation performance on CTPA-derived images. The more vessels were included in the input images, the better the model performed. Each separate investigated vascular region, from the periphery to the central areas, produced similar classification performances for CPE (AUROCs 0.69–0.73). However, the most proximal vascular region seemed to be more relevant (Q1 AUROC 0.85) than the other regions (AUROCs 0.77–0.79) for CTEPH *versus* non-CPE classification. Retraining the segmentation model to include proximal vasculature would facilitate the automation of the proposed analysis process.

## Data Availability

Data supporting this study cannot be made available due to privacy and legal reasons. The trained deep learning models are available from the corresponding author upon a reasonable request.

## Abbreviations

2D: Two-dimensional
3D: Three-dimensional
APE: Acute pulmonary embolism
AUROC: Area under the receiver operating characteristic curve
CNN: Convolutional neural network
CPE: Chronic pulmonary embolism
CT: Computed tomography
CTEPH: Chronic thromboembolic pulmonary hypertension
CTPA: CT pulmonary angiography
MIP: Maximum intensity projection
PE: Pulmonary embolism
Q1, Q2, Q3, Q4: Pulmonary quartile layers (from hilum to periphery) dividing the segmented lung volume into four equal-volume regions
RV/LV: Right ventricle to left ventricle ratio
